# *InternationaL cross-sectIonAl and longItudinal assessment on aSthma cONtrol* in European adult patients - the LIAISON study protocol

**DOI:** 10.1186/1471-2466-13-18

**Published:** 2013-03-25

**Authors:** Fulvio Braido, Guy Brusselle, Eleonora Ingrassia, Gabriele Nicolini, David Price, Nicolas Roche, Joan B Soriano, Heinrich Worth

**Affiliations:** 1 Allergy and Respiratory Diseases Clinic, University of Genoa, IRCCS-AOU San Martino, Genoa, Italy; 2 Department of Respiratory Medicine, Ghent University Hospital and Ghent University, Ghent, Belgium; 3 Chiesi Farmaceutici S.p.A, Via Palermo 26/A, Parma, 43122, Italy; 4 Centre of Academic Primary Care, University of Aberdeen, Aberdeen, UK; 5 Service de Pneumologie et Réanimation, Hôtel-Dieu, Groupe Hospitalier Cochin-Broca-Hôtel-Dieu, Assistance Publique-Hôpitaux de Paris, Université Paris Descartes, Paris, France; 6 Programme of Epidemiology and Clinical Research, Fundació Caubet-CIMERA Illes Balears, Recinte Hospital Joan March, Mallorca, Illes Balears, Spain; 7 Medical Department I, Klinikum Fürth, Fürth, Germany

**Keywords:** Asthma control, ACQ, LIAISON, Observational study, Quality of life, Patient reported outcomes

## Abstract

**Background:**

According to international guidelines, the goal of asthma management is to achieve and maintain control of the disease, which can be assessed using composite measures. Prospective studies are required to determine how these measures are associated with asthma outcomes and/or future risk. The ‘*InternationaL cross-sectIonAl and longItudinal assessment on aSthma cONtrol (LIAISON)*’ observational study has been designed to evaluate asthma control and its determinants, including components of asthma management.

**Methods/design:**

The LIAISON study will be conducted in 12 European countries and comprises a cross-sectional phase and a 12-month prospective phase. Both phases will aim at assessing asthma control (six-item Asthma Control Questionnaire, ACQ), asthma-related quality of life (Mini Asthma Quality of Life Questionnaire, Mini-AQLQ), risk of non-adherence to treatment (four-item Morisky Medication Adherence Scale, MMAS-4), potential reasons for poor control, treatment strategies and associated healthcare costs.

The cross-sectional phase will recruit > 8,000 adult patients diagnosed with asthma for at least 6 months and receiving the same asthma treatment in the 4 weeks before enrolment.

The prospective phase will include all patients with uncontrolled/poorly controlled asthma at the initial visit to assess the proportion reaching control during follow-up and to examine predictors of future risk. Visits will take place after 3, 6 and 12 months.

**Discussion:**

The LIAISON study will provide important information on the prevalence of asthma control and on the quality of life in a broad spectrum of real-life patient populations from different European countries and will also contribute to evaluate differences in management strategies and their impact on healthcare costs over 12 months of observation.

**Trial registration:**

ClinicalTrials.gov identifier, NCT01567280.

## Background

Asthma is a serious global health problem with an increasing prevalence worldwide. People of all ages are affected by this chronic airways disorder that, when uncontrolled, can place severe limits on daily life and is sometimes life threatening or even fatal [1]. A consensus recently stated that there are 300-million people suffering asthma worldwide [2]. Very recently, the costs of persistent asthma have been estimated as EURO 19.3-billion in the whole European population aged from 15 to 64 years, with a mean total cost per patient ranging from EURO 509 in controlled asthma up to EURO 2,281 in uncontrolled asthma [3].

Asthma control is a central focus of the Global Initiative for Asthma (GINA) Guidelines [1], in which clinicians are encouraged to concentrate on its assessment based on the clinical manifestations of disease: symptoms, lung function and the presence or history of exacerbations [4]. Since 2006, GINA guidelines recommend to classify patients into controlled, partly controlled and uncontrolled asthmatics, and highlight that the best way to achieve asthma control is through inhaled anti-inflammatory therapy, monitoring and asthma education [1]. The assessment of asthma control should include not only control of the clinical manifestations but also control of the expected future risk to the patient such as exacerbations, accelerated lung-function decline and side effects of treatments [1].

Treatment of asthma should aim at achieving and maintaining disease control for prolonged periods with a minimum amount of medications, with due regard to the tolerability of treatment, potential for adverse effects, and costs. Effective therapies are now available, and allow attaining asthma control in the majority of patients in randomised controlled trials [5].

However, the proportion of patients lacking asthma control remains high in both adults and children, reflecting a significant gap between what treatments can achieve and the real-life situation [6-10], even in patients receiving regular treatment with inhaled corticosteroids [11,12].

Well-validated self-assessment questionnaires have been developed to monitor the level of asthma control, such as the Asthma Control Questionnaire (ACQ) [13] and the Asthma Control Test (ACT) [14]. These instruments measure asthma symptoms, limitation of activities and need for rescue medication, and have been used in most of the recently published surveys on asthma control.

Country-specific or multinational studies based on the ACQ or the ACT have shown an uneven situation of the asthma control in Europe. In a recent study carried out in the Netherlands [15], the percentage of patients with partly controlled or uncontrolled asthma evaluated with the ACQ was 35.5% and 27.0%, respectively. In another study performed in five European countries [16], approximately half of asthmatic subjects were not well controlled according to the ACT score, and no substantial improvement was found in a more recent survey conducted in the same countries [17]. Conversely, a recent observational study carried out in Italy [18] showed that only 15.8% and 19.8% of patients referred to respiratory medicine centres had partly controlled or uncontrolled asthma, respectively, based on the ACT score. These results confirm previous evidence from a survey conducted in Italy showing that 64.7% of patients with asthma are well controlled [19].

Other studies have evaluated the level of asthma control using different methods of assessment, such as patients’ perception of symptoms [6,20], a questionnaire based on asthma symptoms and recent history [21], and the GINA classification of controlled, partly controlled and uncontrolled asthma [12]. Overall, the results of these studies indicated a suboptimal level of asthma control and variability in the prevalence of controlled patients among European countries [12] or worldwide macro-areas [6].

Most of the observational studies performed until now comprised relatively small populations unrepresentative of the asthmatic population of the countries in which they were performed. In addition, they were mainly based on a cross-sectional design, which does not allow assessing the level of asthma control over time and the impact of adherence to treatment. Furthermore, the limitations due to heterogeneity among methods for assessment of asthma control such as telephone interviews, web-based questionnaires or postal questionnaires, do not allow reaching firm conclusions on patients’ attitudes to asthma management, adherence, level of asthma control and its impact on patients’ quality of life in Europe. The identification of major reasons for a suboptimal asthma control can help the physician to optimise asthma management and the patient to improve his/her perception of the disease.

Based on this background, the ‘*InternationaL cross-sectIonAl and longItudinal assessment on aSthma cONtrol (LIAISON)*’ study has been designed to include a cross-sectional phase and a 12-month prospective phase in order to estimate the level of asthma control in real life, its determinants and its changes during a 1-year follow up.

## Objectives

Table 1 summarises the primary and secondary objectives of the study. The primary objectives of the cross-sectional phase are to evaluate the proportions of patients with controlled, partly controlled and uncontrolled asthma, and to assess the health-related quality of life and the factors associated with asthma control in a real-life population of asthmatic patients.

**Table 1:** Primary and secondary objectives to be investigated

	**Cross-sectional phase**	**Longitudinal phase**
**Primary objectives**	• Prevalence of patients with controlled or uncontrolled/partly controlled asthma	• Proportions of patients with controlled, partly controlled and uncontrolled asthma after 12 months from the cross-sectional phase visit
	• Health-related quality of life	• Proportion of patients with uncontrolled/partly controlled asthma switching to controlled asthma after 12 months from the cross-sectional phase visit
	• Factors associated with asthma control	• Changes in quality of life after 12 months
		• Factors associated with the gain of asthma control
**Secondary objectives**	• Proportion of asthmatic smokers and their level of asthma control	• Association between (current) level of asthma control and (future) risk of exacerbations
	• Antiasthmatic therapies	• Relation between change in asthma control and change in rate of exacerbations during the longitudinal phase (including stratified analyses according to GINA treatment level)
	• Medication adherence	• Antiasthmatic therapies
	• Healthcare costs over 3 months before the cross-sectional phase visit	• Proportion of patients with uncontrolled/partly controlled asthma that reach control after 3 and 6 months from cross-sectional phase visit
	• Rate of severe exacerbations in the last 12 months before the cross-sectional phase visit	• Medication adherence
	• Reasons for poor control according to the Investigators’ and the patients’ opinion	• Healthcare costs
	• Lung function parameters, if available	• Rate of severe exacerbations and the time to first severe exacerbation
		• Reasons for poor control according to the Investigators’ and the patients’ opinion
		• Lung function parameters, if available

The primary objectives of the prospective phase (which will include only patients with uncontrolled and partly controlled asthma classified according to the six-item ACQ score) are to evaluate the proportions of uncontrolled/partly controlled patients reaching asthma control after 12 months from the cross-sectional phase visit, to assess the health-related quality of life and the factors associated with gain of asthma control as well as predictors of those who are at future risk of exacerbations.

## Methods/design

### Study design

**Figure 1:**
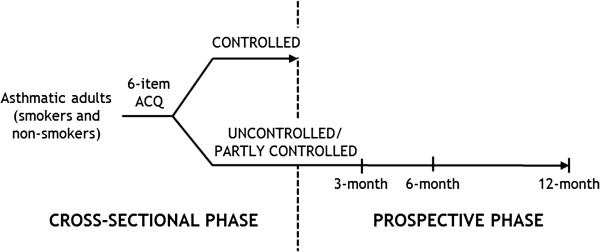
Study design.

Figure 1 shows the design of the cross-sectional phase and the prospective phase of the study. Subjects satisfying entry criteria will be evaluated in the cross-sectional phase of the study. Asthmatic patients with uncontrolled/partly controlled asthma will be followed for the 12-month prospective phase. Follow-up visits will take place approximately 3, 6 and 12 months after the cross-sectional phase visit.

### Study population

The study population will include approximately 8,150 patients attending about 160 outpatient hospitals or General Practice clinics distributed across 12 European countries (Austria, Belgium, France, Germany, Greece, Hungary, Italy, the Netherlands, Poland, Spain, Turkey and the United Kingdom). At least 400 patients in about eight centres will be enrolled in each participating country. Consecutive patients visiting the centre during the estimated 12-month recruitment period will be enrolled.

Male and female adult (aged ≥18 years) patients with a clinical diagnosis of asthma (according to GINA guidelines and confirmed by a chest physician) for at least 6 months, and receiving the same antiasthmatic drugs in the last 4 weeks before enrolment, will be eligible for study participation after signing the informed consent.

Patients participating in a clinical trial within the previous 4 weeks or patients suffering from conditions and illnesses that might interfere with the study purpose, according to the investigator’s evaluation, will be excluded from the study.

### Outcome measures

Information on demographic data, smoking habits, occupational status, professional exposure to asthma risk factors/triggers, concomitant diseases and therapies and asthma history will be collected during the first visit. Due to the observational nature of the study, the spirometry is not included in the study procedures but it will be performed according to the doctor’s evaluation. If available, the lung function parameters will be collected in the case report form. The following self-administered tools will be used for the assessment of asthma control, quality of life and adherence to therapy.

#### Six-item asthma control questionnaire (ACQ)

The six-item ACQ [13] includes a measure of the top five asthma symptoms (woken at night by symptoms, day-time symptoms, limitation of daily activities, shortness of breath and wheeze) and the use of quick-relief bronchodilators. The asthma control level will be evaluated according to the following thresholds: controlled asthma: ACQ score ≤ 0.75; partly controlled asthma: 0.75 < ACQ score <1.5; uncontrolled asthma: ACQ score ≥ 1.5.

As long as many factors are to be considered when a symptom-based approach is being used to achieve optimal disease control [22]*,* the possible reasons for poor control (e.g. comorbidities, seasonal worsening, depression, etc.) will be collected both according to the doctors’ and patients’ opinions using a multiple choice system from a drop down list.

#### Mini asthma quality of life questionnaire (mini-AQLQ)

The mini-AQLQ [23] has been developed to measure the impact of asthma and its treatment that are most troublesome to adults with asthma according to the patient’s perspective and contains 15 questions in four domains: symptoms, activity limitation, emotional function and environmental stimuli. The overall score ranges from 7 (indicating no impairment due to asthma) to 1 (indicating a severe impairment due to asthma).

#### Four-item morisky medication adherence scale (MMAS-4)

The MMAS-4 is a validated scale that estimates the risk of medication non-adherence and consists of four items assessing reasons for non-adherence: forgetfulness, carelessness, feeling better and feeling worse. The Morisky score ranges between 0 (highly adherent) and 4 (highly non-adherent) [24,25].

### Pharmacological therapies

Due to the observational design of the study, antiasthmatic treatments prescribed to patients during the study will be at the discretion of the study physicians according to their clinical judgment and local standards. Antiasthmatic therapies will be recorded in terms of active ingredient, dosage, duration and method of administration. Adverse drug reactions (ADRs) will be recorded for the entire study duration according to the local laws of each country.

### Use of healthcare resources and exacerbations

The number of outpatient visits, hospitalisations and emergency department visits due to asthma will be recorded in order to relate the use of healthcare resources to the level of asthma control.

Information on the number of severe exacerbations, defined as the deterioration in asthma resulting in a hospitalisation or an emergency room visit or the need for systemic steroids for more than 3 days because of asthma, will also be collected [26].

### Data management

Clinical data will be recorded via Electronic Data Capture (EDC) using the HyperSuite-Hypernet XMR® system, an electronic CRF (eCRF). Paper questionnaires (six-item ACQ, mini AQLQ and MMAS-4) filled in by patients during the clinic visits will be entered into the clinical database by independent data-entry operators.

### Sample size

Based on available data from the literature, the proportion of patients with controlled asthma at initial visit (defined as an ACQ score ≤ 0.75) is expected to be about 37.5% [15]. Considering that patients with uncontrolled and partly controlled asthma at the cross-sectional phase visit will be included in the prospective phase, the percentage of patients with controlled asthma at month 12 is expected to be approximately 45% [27].

By enrolling 8,150 patients, it is expected that 5,094 patients should have uncontrolled/partly controlled asthma at the cross-sectional phase visit. The proportion of patients expected to drop out, for any reason, during the 12-month longitudinal phase is 20%. Therefore, 4,075 patients should be evaluable at month 12, which allows estimating the expected 45% of patients reaching asthma control at the end of longitudinal phase with a precision of ± 1.5% (two-sided 95% CI).

With regard to the assessment of quality of life, a standard deviation (SD) for the Mini AQLQ overall score of approximately 1.21 units can be estimated from the literature [28]. If 4,075 patients are evaluable for the analysis of the Mini AQLQ overall score from the cross-sectional phase visit, then the distance from the boundaries of the two-sided 95% CI to the point estimate will be 0.037 units. Therefore, considering the expected patients reaching asthma control at the end of the longitudinal phase, the distance from the boundaries of the two-sided 95% CI to the point estimate will be 0.055 units.

### Statistical analysis

All recorded variables will be tabulated using summary statistics for continuous variables and frequency tables (absolute and relative) for categorical variables. All outcomes will be stratified by country.

Logistic regression analysis will be used to analyse binary variables (e.g. the association between the asthma control level and quality of life in the cross-sectional phase). Models will include confounding factors if identified by the exploratory analysis. As measures of association between the response variable (e.g. the asthma control level) and the independent variables, the odds ratios (ORs) with the relative 95% CI along with the p-value will be reported. For continuous variables, the estimated OR will be expressed for a change of *c* units in the covariate.

The analyses of the results of the longitudinal phase will be conducted using mixed-effect models (SAS Proc MIXED) to estimate the means of the changes in the scores over time from the cross-sectional phase visit.

The healthcare costs will be evaluated by descriptive analysis, considering the time frames of 3 months before the cross-sectional phase visit and the longitudinal phase period.

The number of severe exacerbations in the last 12 months before the cross-sectional phase visit and the number of severe exacerbations per patient/year at each visit will be descriptively analysed overall and by level of asthma control. The rate of severe exacerbations per patient/year will be calculated as the total number of experienced severe exacerbations over the longitudinal phase on total number of days of observation of patients at risk. The time to first asthma severe exacerbation during the longitudinal phase will be analysed with the Kaplan-Meier survival analysis, and their significance by the log-rank test. Predictors of exacerbations will be analysed by means of a multivariate logistic model. A stepwise selection approach will be used to identify the most significant prognostic factors and to eliminate the unimportant ones. Using this methodology, a final predictive model containing just the important variables will be created.

### Ethics

This trial will be conducted in compliance with the Declaration of Helsinki (1964 and amendments), Good Clinical Practices (GCP) and all relevant local laws and regulations. Patients will give their written informed consent prior to the start of any study-related procedure and the study protocol has been approved by the reference Ethics Committee of each participating site (Additional file 1: Appendix).

## Discussion

The aim of the LIAISON study is to estimate the level of asthma control and quality of life in a European real-life setting and their evolution during 1 year, using validated self-administered tools such as the six-item ACQ and Mini-AQLQ. Further objectives are: to evaluate the reasons for lack of asthma control, the medication adherence, the impact of pharmacological treatment, the number of severe exacerbations and healthcare resources use.

Several real-life asthma studies have been reported elsewhere but the variability in patients’ samples due to current treatments, the geographical location and the criteria for exclusion from study participation has led to different outcomes in terms of prevalence of asthma control. Moreover, it is difficult to compare the results of these studies due to the different methods used in data collection, which included patient self- or web-administered questionnaires, office-based or hospital-based physician consultations [18].

With the inclusion of more than 8,000 patients in the cross-sectional phase and of more than 4,000 in the longitudinal phase of the study, the LIAISON study will be the largest naturalistic study ever performed in 12 European countries to investigate asthma control and the impact of asthma control on quality of life and future risk of exacerbations. Furthermore, the longitudinal phase of the study will provide important information on the adequacy and effects of management strategies implemented in each country to reach control in the following year.

Previous multinational studies carried out in Europe have shown important differences among countries in the level of asthma control. Rates of not well-controlled asthma ranged from 65% in Germany to 40% in Spain [16] or from 63% in Italy to 41% in France [17]. Among ICS users, the prevalence of uncontrolled asthma ranged from 20% in Iceland to 67% in Italy [12]. Differences in symptoms’ control between Western and Central/Eastern Europe, with somewhat better outcomes in Western countries, were also reported [6]. In most of these studies, assessments were performed by phone- or mail-transmitted questionnaires based on patient perception of asthma control and severity of symptoms [6,12,20], or were internet-based with data obtained from the European National Health and Wellness Survey [16,17]. Moreover, all the above studies were cross-sectional and, therefore, gave no information on the prospective monitoring of patients with poor asthma control.

In the LIAISON study, the assessments will be performed during patient visits at the clinics, thus allowing a real-life asthma management approach based on disease monitoring. It has been suggested that monitoring outcomes and taking appropriate action through regular visits may improve current levels of asthma control. Behavioural factors such as smoking and non-adherence may reduce the efficacy of treatment and patient perceptions influence these behaviours. Under-treatment may also be related to patient underestimation of the significance of symptoms, and lack of awareness of achievable control [14].

We chose the 6-item ACQ for the evaluation of asthma control because it is applicable to all adults with asthma, is considered reliable and reproducible. It has been fully validated for use in both clinical practice and clinical trials, and the minimum clinically important difference has been established [13]. The ACQ also has strong discriminative properties, i.e. it is able to detect small differences between patients with different levels of asthma control and it is very sensitive to within-patient changes in asthma control over time [27]. The overall population included in the LIAISON study will be representative of the European real-life setting. A sample size of at least 400 patients enrolled in at least eight centres in each country will also allow reliable country-specific analyses of data collected in a number of sites that are representative of the entire National territory in all involved countries [6]. Previous reports have included a limited number of sites [12] or a smaller number of patients in some countries than in others [6], while other studies have been performed in sites with non-homogeneous distribution in the same country [21].

Asthmatic smokers and pregnant women, who are usually excluded from randomised controlled trials (RCT) in asthma, will be included in the LIAISON study in order to obtain a large sample that is as representative as possible for the real-life asthmatic population in Europe. Smoking is a critical factor associated with increased risks of not achieving control, excess mortality, asthma attacks and exacerbations [22,29]. Within all obstructive respiratory disorders, asthmatics who smoke represent a substantial portion that increases with age [30]. Therefore, the non-exclusion of smokers in previous observational studies [16,18] may in part explain the increased frequency of poor asthma control in these studies compared to rates observed in RCT, and may have led to differences among countries. One weakness of this study is the requirement for questionnaire completion and patient consent, which will tend to bias the data towards those managed in good clinical settings. On the other hand, the recruitment of consulting patients could tend to bias towards those who are uncontrolled. Other important endpoints of the study will be the description of the reasons for poor control (from both the patient’s and investigator’s perspectives), the evaluation of patient adherence to antiasthmatic therapy and the impact of suboptimal asthma control on healthcare costs. There are limited data available on the cost-effectiveness of treatment strategies aimed at different levels of asthma control [31,32] and other local studies investigating cost-effectiveness of asthma treatment strategies driven by different target levels of asthma control are ongoing [33].

To our knowledge, the LIAISON study will be the first to include both a cross-sectional and a longitudinal phase to assess asthma control and quality of life in a large population from a number of European countries. Therefore, the study will allow obtaining pan-European data while evaluating among-country differences in care with consistency and its impact from an economic perspective on the different national healthcare systems.

In conclusion, it is expected that the multinational LIASON study will provide novel data on the level of asthma control and quality of life in clinical practice in Europe. The results of the study will contribute to understanding the reasons for poor asthma control and to evaluate the proportion of patients with uncontrolled or partly controlled asthma who achieve asthma control in a 1-year observation period. Moreover, the study will provide data on adherence to treatment, number of exacerbations and healthcare resource use, together with insights into the impact of pharmacological treatment on both clinical and pharmacoeconomic outcomes.

## Competing interests

In the past 5 years, FB received fees for speaking, organising education or consulting from GlaxoSmithKline, AstraZeneca, Menarini, Chiesi, Abbott, Sigma-Tau, Novartis, MSD.

GB has, within the last 5 years, received honoraria for lectures from AstraZeneca, Boehringer-Ingelheim, Chiesi, GlaxoSmithKline, Novartis, Pfizer and UCB; he is a member of advisory boards for AstraZeneca, Boehringer Ingelheim, GlaxoSmithKline and Novartis.

EI and GN are employees of the sponsor company.

DP has consultant arrangements with Almirral, Astra Zeneca, Boehringer Ingelheim, Chiesi, GlaxoSmithKline, Merck, Mundipharma, Medapharma, Novartis, Napp, Nycomed, Pfizer, Sandoz and Teva. He or his research team have received grants and support for research in respiratory disease from the following organisations in the last 5 years: UK National Health Service, Aerocrine, AstraZeneca, Boehringer Ingelheim, Chiesi, GlaxoSmithKline, Merck, Mundipharma, Novartis, Nycomed, Orion, Pfizer, and Teva. He has spoken for: Almirral, AstraZeneca, Activaero, Boehringer Ingelheim, Chiesi, Cipla, GlaxoSmithKline, Kyorin, Novartis, Merck, Mundipharma, Pfizer and Teva. He has shares in AKL Ltd, which produces phytopharmaceuticals. He is the sole owner of Research in Real Life Ltd and its subsidiary social enterprise Optimum Patient Care.

In the past 5 years, NR received (i) fees for speaking, organising education, or consulting from Altana Pharma-Nycomed-Takeda, AstraZeneca, Boehringer Ingelheim, Chiesi, GlaxoSmithKline, MEDA, MSD-Chibret, Mundipharma, Novartis, Pfizer, Teva; (ii) research grants from Nycomed, Boehringer Ingelheim, Pfizer and Novartis.

JS received pharmaceutical company grants from GSK in 2011 and Chiesi in 2012 via his home institution, and also participated in speaking activities, industry advisory committees or other related activities sponsored by Almirall, Boehringer Ingelheim, Pfizer, Chiesi, GlaxoSmithKline and Novartis during the period 2010–2012.

HW has consultant arrangements with Almirall, Berlin Chemie, Bionorica, Chiesi, Klosterfrau, Munipharma, Novartis, Nycomed. He has spoken for Almirall, AstraZeneca, Chiesi, Boehringer, Klosterfrau, Novartis, Nycomed and Pfizer. In the past 5 years, research was supported by Novartis, Klosterfrau and Nycomed.

## Authors’ contributions

All authors contributed to the design of the study and have read and approved the final manuscript.

## Pre-publication history

The pre-publication history for this paper can be accessed here:

http://www.biomedcentral.com/1471-2466/13/18/prepub

## Supplementary Material

Additional file 1 **Appendix.** Ethics Committees that evaluated the LIAISON study protocol. Click here for file
