# Supplementary material for: InternationaL cross-sectIonAl and longItudinal assessment on aSthma cONtrol in European adult patients - the LIAISON study protocol
Source: BMC Pulm Med. 2013 Mar 25;13:18. doi: 10.1186/1471-2466-13-18 (PMC3623774; doi:10.1186/1471-2466-13-18)
Supplement: Additional file 1 — Appendix. Ethics Committees that evaluated the LIAISON study protocol. [file 1471-2466-13-18-S1.pdf]

APPENDIX

Ethics Committees that evaluated the LIAISON study protocol.

AUSTRIA

CENTRAL EC: Ethikkommission der Stadt Wien, WIEN.

BELGIUM

CENTRAL EC: UZ Gent, GENT.

LOCAL ECs: CUB Hôpital Erasme, BRUXELLES; CHU Vésale, MONTIGNY LE TILLEUL; CHU Charleroi UZ Gent, GENT; VZW Emmanus, MECHELEN; CHR de la Citadelle, LIÈGE; Ethisch Comité van het Jan Yperman Ziekenhuis, LEPER; Hôpitaux Iris Sud, BRUXELLES.

FRANCE

Comité Consultatif sur le Traitement de l'Information en Matière de Recherche dans le Domaine de la Santé (CCTIRS).

GERMANY

CENTRAL EC: Ärztekammer des Saarlandes Ethikkommission, SAARBÜCKEN.

LOCAL ECs: Ethik-Kommission der Ärztekammer Westfalen-Lippe und der Medizinischen Fakultät der Westfälischen Wilhelms-Universität Münster, MÜNSTER; Landesärztekammer Baden-Württemberg Ethikkommission, STUTTGART; Ethik-Kommission bei der Ärztekammer Berlin, BERLIN; Ethikkommission der Ärztekammer Nordrhein, DUSSELDORF; Ethikkommissionen bei der Ärztekammer Schleswig-Holstein, BAD SEGEBERG; Ethikkommission bei der Sächsischen Landesärztekammer, DRESDEN; Ärztekammer Niedersachsen Ethikkommission, HANNOVER; Landesärztekammer Thüringen Ethikkommission, JENA; Landesärztekammer Brandenburg Ethikkommission, COTTBUS.

GREECE

LOCAL ECs: Sismanoglio-Amalia Fleming General Hospital of Attica (Legal Entity of Public Law), MAROUSI; Sotiria Chest Diseases General Hospital of Athens, ATHENS; Metropolitan" Hospital, NEO FALIRO; Amalia Fleming General Hospital of Melissia, MELISSIA; Hygeia Hospital, MAROUSI.

HUNGARY

CENTRAL EC: ETT TUKEB (Egészségügyi Tudományos Tanács, Tudományos és Kutatásetikai Bizottság), BUDAPEST.

ITALY

LOCAL ECs: Comitato Etico dell'Azienda Ospedaliera - Ospedali Riuniti Villa Sofia-Cervello di Palermo, PALERMO; Comitato Etico per la Sperimentazione Clinica della Provincia di Verona, VERONA; Comitato Etico per la Sperimentazione Clinica dei Medicinali dell'Azienda Ospedaliero-Universitaria Carreggi di Firenze, FIRENZE; Comitato Etico Scientifico dell'Azienda Ospedaliera - Ospedale S. Carlo Borromeo di Milano, MILANO; Comitato per la Sperimentazione Clinica dei Medicinali dell'Azienda Ospedaliero-Universitaria di Pisa, PISA; Comitato Etico dell'Azienda Ospedaliero-Universitaria Policlinico Paolo Giaccone dell'Università degli Studi di Palermo, PALERMO; Comitato Etico dell'Azienda Ospedaliero-Universitaria Mater Domini di Catanzaro, CATANZARO; Comitato Etico Indipendente per la Valutazione delle Sperimentazioni Cliniche dei Medicinali dell'Azienda Ospedaliera Santa Maria degli Angeli di Pordenone , PORDENONE; Comitato Etico dell'Azienda Ospedaliero-Universitaria S. Martino di Genova,

GENOVA; Comitato Etico dell'Azienda Ospedaliera S. Anna e S. Sebastiano di Caserta, CASERTA; Comitato Etico dell'Azienda Ospedaliera Policlinico Consorziale di Bari, BARI; Comitato Etico della ASL Napoli 2 Nord di Pozzuoli, POZZUOLI (NA); Comitato Etico della ASL 4 di Teramo, TERAMO; Comitato Etico dell'Azienda Ospedaliera Ospedali Riuniti S. Giovanni e Ruggi d'Aragona, SALERNO; Comitato Etico Locale per la Sperimentazione Clinica dell'Azienda Ospedaliera Luigi Sacco di Milano, MILANO; Comitato Etico dell'Azienda Ospedaliera della Provincia di Lodi, LODI; Comitato Etico per la Sperimentazione Clinica della Provincia di Padova, PADOVA; Comitato Etico della ASL RM/B di Roma, ROMA; Comitato Etico dell'Azienda Ospedaliero-Universitaria Ospedali Riuniti di Foggia, FOGGIA; Comitato Etico della ASL TO/2 di Torino, TORINO; Comitato Etico per la Sperimentazione Clinica dei Medicinali dell'Azienda Sanitaria di Firenze, FIRENZE; Comitato Etico della ASL di Cagliari, CAGLIARI.

POLAND

CENTRAL EC: Lubelska Komisja Bioetyczna przy Okręgowej Izbie Lekarskiej, LUBLIN.

SPAIN

CENTRAL EC: Ethics Committee of Islas Baleares, PALMA DE MALLORCA.

LOCAL ECs: CEIC Hospital de Jerez, JEREZ DE LA FRONTERA; Cap Unitat Suport al CEIC - Secretària tècnica CEIC HUVH - Vall d'Hebron Institut de Recerca (VHIR), BARCELONA; Unidad Administrativa CEIC Instituto de Investigación Hospital 12 de Octubre (i+12), MADRID; CEIC Hospital Reina Sofía de Córdoba, CÓRDOBA; CEIC Hospital Universitario Carlos Haya, MÁLAGA; CEIC Servei de Recerca i Assaigs Clínics. Fundació Sant Joan de Déu, ESPLUGUES DE LLOBREGAT (BARCELONA); CEIC Hospital Universitari Germans Trias i Pujol, BADALONA (BARCELONA); CEIC Hospital Universitario San Cecilio, GRANADA; CEIC Teknon - Unidad de Gestión de Ensayos Clínicos - Unitat de Gestió d'Assajos Clínics, BARCELONA; CEIC Fundació de Gestió Sanitària Hospital de la Santa Creu i Sant Pau, BARCELONA; CEIC Hospital Gregorio Marañón, MADRID; CEIC Hospital Ramón y Cajal, MADRID; CEIC Hospital Universitario Puerta de Hierro Majadahonda, MAJADAHONDA (MADRID); Secretària de Recerca i CEIC Consorci Hospitalari de Vic, VIC (BARCELONA); Ethics Committee of Islas Baleares, PALMA DE MALLORCA; Secretaria Administrativa del CEIC del CSdM Hospital de Mataró, MATARÓ (BARCELONA); CEIC de la Corporació Sanitària Parc Taulí, SABADEL (BARCELONA); Secretaria Técnica CEIC - Fundación para la Investigación Biomédica del Hospital Universitario La Princesa, MADRID.

THE NETHERLANDS

CENTRAL EC: METC Isala klinieken, ZWOLLE.

LOCAL ECs: Gelre Ziekenhuis Zutphen Lokale METC, ZUTPHEN; METC Isala klinieken, ZWOLLE; Orbis Medisch Centrum Sittard, SITTAN-GELEEN; Academisch Medisch Centrum Universiteit van Amsterdam Medisch Ethisch Toetsingscommissie, AMSTERDAM; Diakonessenhuis Utrecht, ULTRECHT; MEC St. Anna Ziekenhuis Geldrop, GELDROP; Laurentius Ziekenhuis Roermond, ROERMOND; Ikazia ziekenhuis Rotterdam, ROTTERDAM.

TURKEY

CENTRAL EC: Çanakkale Onsekiz Mart University Medical Faculty Clinical Researches Ethics Committee, ÇANAKKALE.

UNITED KINGDOM

CENTRAL EC: NHS Health Research Authority - National Research Ethic Service - NRES North West GM East, MANCHESTER.

LOCAL ECs: NHS Cornwall and Isles of Scilly, TRURO; NHS Leicester County and Rutland - Leicester, Leicestershire & Rutland Primary Care Research Office, LEICESTER; NHS Devon - NHS Plymouth - Torbay Care Trust - NHS Devon Commissioning Headquarters, EXTER; Sussex NHS Research Consortium, WORTHING.
